# The Study of Emotional Effects of Digitalised Work: The Case of Higher Education in the Sustainable Development

**DOI:** 10.3390/ijerph19010576

**Published:** 2022-01-05

**Authors:** Iwona Staniec, Dominika Kaczorowska-Spychalska, Magdalena Kalinska-Kula, Nina Szczygiel

**Affiliations:** 1 Department of Management, Lodz University of Technology, Piotrkowska 266, 90-924 Łódź, Poland; 2 Department of Marketing, Faculty of Management, University of Lodz, Matejki 22/26, 90-237 Łódź, Poland; dominika.spychalska@uni.lodz.pl (D.K.-S.); magdalena.kalinska@uni.lodz.pl (M.K.-K.); 3 GOVCOPP, Department of Economics, Management, Industrial Engineering and Tourism, University of Aveiro, 3810-193 Aveiro, Portugal

**Keywords:** technological innovation in education, smart working, impact assessment on emotions, communication, risk management, COVID-19, Poland

## Abstract

This paper reports on the experiences of working with new digital tools along with the experience of new remote work. We explore the emotional experiences of working from home during the first three months of the COVID-19 pandemic and their implications. There were two groups of respondents participating in the study, those who had experience working remotely before the pandemic [digital natives] and those who started working remotely during the pandemic [digital immigrants]. The results show that emotional experiences while working from home do not differ depending on the profession, age, gender, length of experience and from previous remote work. This suggests that the digital natives had to deal with the same emotions as the digital immigrants. The study found that independent external changes determine the growth of competence in employees, in this particular case, to work remotely. Working in conditions that are difficult for everyone obliges employees to cooperate, even across company boundaries, and increases each other’s competencies. In such situations, the management is required to be emotionally involved and closer to the employee.

## 1. Introduction

The COVID-19 pandemic that took the world by surprise in early 2020 has wreaked havoc around the world, closing businesses and changing the way in which entire societies live and work. Voluntary precautionary measures against COVID-19 as well as mandatory government restrictions have resulted in millions of workers being instructed by their employers to start working from home [1]. The pandemic has undoubtedly influenced the introduction of digital technologies in all areas of human activity deeper than any previous global crisis [2], but the area characterised by the greatest change is the implementation of remote work [3].

Within a dozen or so weeks, the COVID-19 pandemic caused a radical change-the transition from traditional work to remote work. Organisations were forced to change the scope of existing technologies and to rapidly transform into remote working mode [3]. Lockdown made people realise that working remotely, using existing technologies, is possible even in the case of work that was previously associated only with traditional work in the office [4].

Remote work in times of crisis has become a key source of economic and organisational resilience [5], however, such a widespread shift has raised concerns-both about continued productivity and the well-being of employees [6]. At the same time, the benefits for employees resulting from regular remote work were noticed, including autonomy as well as time and space flexibility in individual work processes [7]. A large number of purely home workers have incorporated existing technology into their daily routine activities [1].

Today, many journalists, practitioners, and academic commentators speculate that working at home on a large scale will become the “new normal” after the pandemic [8]. Perhaps the workplace mono-model will no longer be sufficient, and employers should look for more creative solutions, such as a hybrid model, which may prove beneficial for both employees and employers [9].

The article is a continuation of the previous reflections and analyses on remote work and as such is part of the ongoing debate on the consequences and challenges of this form of work for employees. In recent years, many scientific works on remote work have been published, but the research undertaken in this area is largely focused on indicating the essence, advantages and limitations of this form of work. Research works on the perspective of social acceptance of remote work and employees’ emotions accompanying the process of implementing these solutions are published less frequently. It is understandable that in pandemic circumstances it is important to develop a response in this regard. The theoretical gap was identified in the field of recognizing the emotional determinants of remote work in a situation of coercion resulting from the COVID-19 pandemic. In this context, the research gap concerns in particular the lack of up-to-date research among representatives of the academic community. 

Against the background of the identified theoretical gap, the main goal of the article was to identify the perception of remote work and to define experiences related to remote work in the COVID-19 environment, with particular emphasis on the role of emotions as the main determinant of this process.

The following structure of the work was adopted for its implementation: the literature review on the theoretical basis of remote work with an indication of the hypotheses, methodology of the research, presentation of the obtained results with the limitations, discussion and identification of theoretical and practical implications.

## 2. Literature Review

### 2.1. Remote Work-Specificity, Advantages and Disadvantages

Work performed outside of the spaces provided by the employer has been known under many names, such as telecommuting, telework, virtual work, remote work and distributed work [10]. Over time, the search for a universally accepted definition of telework has been a source of serious disputes and debates [11], however, it can undoubtedly be assumed that remote work has some features that distinguish it from traditional office work. The primary factor is physical distance which is managed through reliance on communication technology. Another feature that defines remote work is limited supervision. Finally, working remotely requires interpersonal connections with other people [or teams of people] in the organisation [12]. The definitions presented in the literature review highlight aspects related to remote work, such as physical distance, freedom of location, the use of information and communication technologies, organisational flexibility, and the interdependence of its members. They reflect holistically the multithreading of issues related to enabling the integrated functioning of the entire organisation, regardless of the distance and location of its individual employees.

When analysing how the role of remote work has evolved in society and business over time, two main stages should be indicated-the pre-COVIDstage, i.e., optional remote work, and the COVIDstage, i.e., a rapid transformation in remote work [3]. 

Some flexible working practices, such as part-time work, have been widely adopted over the past few decades, although initially, employers were rather reluctant to implement homework due to some uncertain benefits and inadequate technology [13]. Over time, offering the opportunity to work anywhere and anytime, teleworking has gained favour with scientists and professionals alike. It began to be considered a win-win situation for employees and organisations, thanks to the possibility of lowering costs, motivating employees and creating a work-life balance [14]. This was undoubtedly influenced by the parallel development and implementation of information and communications technology [ICT], which increased the speed and quality of work performed without the need for an employee to be physically present in the organisation and at the same time minimising the associated costs [15]. In addition, the transition from a productive economy to a knowledge-based economy has increased the number of jobs suitable for remote work [16]. Digital technology changed the characteristics of work, allowing for its multi-dimensional fragmentation of administration [increasingly complex employment relationships-direct and subcontracting], time [use of part-time and shift work] and space [smaller and more isolated units of work] [17].

While flexibility is assumed to be a key benefit, allowing employees to better balance their work and home responsibilities [18,19,20], it undoubtedly requires constant balancing between the two areas of employee activity. Increasing professional autonomy can help ease the pressure associated with family responsibilities, assuming that the employee receives or has access to the necessary resources, including cognitive and emotional ones, to facilitate the implementation of specific tasks [21]. Depending on the level of intensity of the undertaken activities [intensive versus extensive work], employees may gain more time for work at the expense of domestic duties and vice versa [20]. However, their wrong configuration will lead to conflicts [Home-to-Work Conflict, Work-to-Home Conflict] [17]. Unfortunately, the ability to perform tasks in the area of work conducted with the use of ICT is not adapted to the employee’s life situation [22], and the lack of a place to work, the lack of appropriate equipment, additional costs that they must incur, the features of platforms or communicators through which they work] will increase the feeling of pressure, emotional and physical fatigue [23,24] and can additionally lead to a feeling of excessive strain [especially in the case of people who are not used to working autonomously] [25]. In the current situation of the COVID-19 pandemic, these feelings will be simultaneously exacerbated by constant fears for the health and safety of their loved ones.

A high level of intensity of tasks performed as part of work using ICT may also increase employees’ dependence on these solutions, increasing their sense of isolation, as a result of weakening ties with colleagues, weakening the level of loyalty and identification with the company and promoting gradual social exclusion [23,26,27]. This will be reflected in the quality of the tasks performed, and the accompanying psychological effects [stress, emotional anxiety, dissatisfaction, etc.] [28] may reduce well-being, motivation to work, and the development of further professional career [29]. 

During the course of the coronavirus pandemic that has spread around the world, it was necessary to quickly, almost immediately move to remote work from all sectors of the global economy and social activity. In response to this need, many innovative systems and services for personal and team remote communication were created, including tools for remote work, such as MS Teams, Skype, or Zoom, which started to be widely and successfully used by millions of users-both employees and clients [3]. Remote work has become the only way to continue the implementation of tasks, and the uncertainty as to the duration of the pandemic has led to its perception as a “new normal” in the daily operation of the organisation [18].

The coronavirus pandemic has forced enterprises to adopt digital transformation. Many organizations have successfully transformed their workflows and the number of employees working remotely tends to increase. The indicated digital transformation processes are closely linked with the development of human capital competencies and digital skills. With the digital transformation processes deepening, the most important issue is the development of relevant competencies, acquiring new knowledge, work organization and management skills. Along with the development of specific digital skills, it is essential that individuals also build up soft skills, such as the ability to communicate with others, work in a team act creatively, be able to start new activities, show initiative and enthusiasm, put forward real aims and try to achieve them [22]. To face challenges and seize opportunities, employees need to develop technical and human skills. People today need to drive their own careers with a commitment to long-life learning and thriving [29].

Due to the COVID-19 pandemic, which forced millions of people employed in various industries and in various positions, to work at home, employees regardless of their knowledge of digital tools and attitude to distance communication, had to rapidly develop digital competencies [4,30]. Therefore, we hypothesise that:

**Hypothesis** **1** **(H1).** *The incremental competence to work remotely during the initial period of COVID-19 was significant regardless of age, position, and experience.*

### 2.2. The Nature of Emotions

Emotions are central to human nature. When triggered, emotions interact with cognition releasing perception, attention, goal setting, decision making, learning and memory systems and physiological reactions [31] and communicate to the world our emotional state of mind. 

We seem to be profoundly emotional: not only do we experience emotions on daily basis, but each day offers plenty of emotional experiences. One study that used a smartphone application to monitor real-time emotions suggested that 90% of the time people feel at least one emotion [32]. Despite the fact that emotions have been examined since ancient times [33], they became a particularly compelling object of study to different scientific disciplines only a few decades ago, the moment in time that corresponded to the rise of neuroscience and the universal dissemination of information and communication technologies. Thousands of research items later, the fundamental question of what emotion is, persists. To illustrate this research panorama, Plutchik [34] enlists 27 different definitions. Probably the greatest paradox in emotion research consists of the fact that activated emotions are very clearly perceived and identified which does not make them any easier to be formally defined.

In an attempt to bridge that gap, Izard [35] asked a number of renowned emotion re-searchers to provide their interpretation of emotion which resulted in the following description: “Emotion consists of neural circuits [that are at least partially dedicated], response systems, and a feeling state/process that motivates and organises cognition and action” [p. 367]. Emotions have also been regarded as short-lived purposive mental reactions to an important life event. They play an important cognitive and social function and allow to direct attention to an event and organise internal resources to prepare a successful response. Additionally, the recognition of emotions is fundamental to human social interactions. Reeve [36] regards emotions as subjective mental states elicited by sensory input generating behavioural output in the form of “feeling-purposive-expressive-bodily” responses [p. 288]. In this understanding, a four-dimensional character of emotions emphasises four distinct but interrelated aspects of emotions. 

Basic emotions, such as fear, disgust, or anger, arise from the subcortical neural structures and pathways and transmit notification conveying urgent information that the body’s well-being or survival might be at risk. Anxiety for instance is a basic aversive emotional state in which an individual is apprehensive about what is about to come. This is different from fear in that it does not have a precise origin and does not raise a specific coping response [a person feels restless and tense about the unknown future developments]. Such reaction is experienced in new, especially unexpected situations to which there has been little or no time to duly prepare and which obliges an individual to change known practices and rely on new tools. This, undoubtedly, induces stress. 

Higher-order emotional states develop from the cortical structures and pathways on the basis of personal experiences and expectations, and social contexts [37]. What normally triggers such emotions is cognitive appraisal, an assessment one makes, often unconsciously, of the significance of the event [38,39] to the beliefs and values of the self [40]. The modern brain relies on the mind-environment nexus and filters the incoming information to respond suitably. Among self-conscious emotions, shame is regarded as an experience of social inferiority, incompetence and worthlessness [41], and followed by the tendency to withdraw or hide [42]. 

The COVID-19 pandemic and the transition to a technology-related form of work that was unknown among many employees induced stress [31,37,41]. Therefore, it is hypothesised that:

**Hypothesis** **2** **(H2).** *Employees experienced negative emotions during this period related to the new situation, unfamiliarity with the tools, feelings of uncertainty*.

### 2.3. Technology Adoption and Emotions

The world is in constant motion. Similar to biological evolution, technology is an evolutionary process in which positive feedback loops, such as cost-effectiveness or speed, increase exponentially over time and the rate of rapid growth itself exponentially increases allowing for incremental growth of societies [43]. The world we live in today is built upon advances of previous generations, those that developed mass production, gave rise to high-level automation and brought technological solutions to daily life. Nowadays, technology feels like it is accelerating. Digital ecosystems are engraved in our daily life. Information flows constantly through billions of digital devices and messages are being delivered to everyone and everywhere all the time [44]. 

However, technology is not natural to everyone. Younger, tech-savvy generations effortlessly navigate the digital space finding the procedural aspects of technology use to be natural and feel at ease adopting new ones. Born in or right before the internet era, they outweigh other generations in most measures of technology adoption [45] and technological literacy plays a pivotal role in their social, academic and professional interactions [46]. A survey on parents’ and teenage children’s attitudes to technology and loneliness revealed that for 51% of the younger subjects the internet was a source of social relations, social support and advice and 64% had never had a negative experience when using technology. Conversely, the parents were much less confident as to the positive impact of technology on their lives [47]. In regard to the internet-loneliness nexus, Nowland and colleagues [48] propose a bi-directional character of the relationship. The internet can be a means to reduce loneliness when it is used to establish new and deepen existing social connections, but not when the digital domain is perceived as an escape from social interactions. A body of research has suggested that the internet can mediate joy and compassion, promote social [re]connectedness and have a positive impact on life satisfaction [49,50]. Yet, this does not necessarily prevent people from suffering technology-related stress. A study by Afifi et al. [51] that examined the impact of media and technology on stress measured by cortisol levels and inflammation measured by interleukin [IL-6] levels, found that the use of technology had the greatest impact on adolescents. Subjects who were often online and had large social networks with whom they interacted frequently were found to have higher levels of cortisol and IL-6. 

In contrast, individuals born before the era of the internet embrace the digital realm at a scale by no way comparable to younger generations. They accept the internet and social media as a new fundamental skill and seek digital literacy training in order to build confidence to access information and services online for social and professional purposes. However, despite advances in technology use, many cannot keep pace. It is highly likely that older professionals would react to the idea of using a new digital solution with mistrust and resistance rather than giving it an enthusiastic reception. Technologies do have their problems and inexperienced users lacking confidence may encounter insufficient safety standards and privacy issues. A theoretical model proposed by Venkatesh [52] posits that behavioural intention to use the technology is determined by perceived ease of use and perceived usefulness, both of which are problematic in older users. Confrontation with the latest technological product can be uncomfortable, intimidating and raise attitudinal barriers to technology acceptance [53], which can generate stress and anxiety. A negative affective reaction towards technology use has been shown to exert a negative influence on attitudes and employee performance [54,55]. Moreover, the pressure to be constantly connected to the stream of updates and messages also seems to have a significant impact on one’s well-being. Persistent notifications may be distractive and decrease concentration and productivity, which can be distressing and engender sleep disorders [56].

If we thought that the proportion of technology-based workplaces was significant, then the outbreak of the COVID-19 pandemic has taken it to another level. While the process of transformation was rather reactive than proactive, a shift to a modern digital working environment seems now well-established. In its original idea, the digital workplace is a holistic system of platforms, tools and business systems [57,58], now integrated into a home’s physical environment. More than ever, we live and work surrounded by technologies with the possibility of allowing for more flexibility [59], but with blurred boundaries between what is home and what is work. Rather than a choice, technology has become an imperative. A recent study conducted in Singapore has found that not only was remote work associated with high levels of stress, but employees working from home also experienced higher levels of stress than those working in the COVID-19 frontline [60]. A survey conducted in the UK context by the University of Kent and the University of Birmingham investigated the impact of the pandemic, the lockdown and working from home on a range of work-life issues. According to the results, on average 44% of the participants experienced pressure [highest among mothers] and 44% felt tense and stressed [61].

The use of technology and social media during the pandemic increased [62] and excessive use of social media was significantly associated with higher anxiety [62]. Technology provides a unique encounter through which we can express and experience different emotional states. Studies have embraced both of these perspectives and have provided empirical evidence on the relationship between digital technology and particular emotions, such as hope, compassion, empathy, or envy [see [63], for more details]. In her work on emotion management, Hochschild [64] explored the most urgent challenges of contemporary societies and argues that 40% of jobs require considerable amounts of emotion management. It is unrealistic to believe we can remove ourselves from our emotions and it is natural to experience them when we face an unknown situation that makes us uncomfortable. One way to cope with emotions is by choosing an event we want to expose ourselves to. While some studies suggest positive home and technology-based work outcomes [65,66], those greatly needed in developing resilience and coping resources in challenging times, vast evidence [57,58,59,60] shows that new situations and health-related threats may cause emotional dissonance. In a study on the link between social media use and health-related outcomes, Bekalu et al. [65] found emotional connections to be associated with lower mental health, social well-being and self-rated health. The same was not confirmed when the use of social communication was routine. 

Therefore, it is hypothesised that:

**Hypothesis** **3** **(H3).** *Employees exhibited emotional dissonance during the analyzed period of the COVID-19 pandemic.*

## 3. Materials and Methods

### 3.1. Study Context and Sample

The study population was employees of higher education institutions [HEIs] in Poland. The survey was administered in two stages. In the first stage, the e-mail addresses of the HEIs were retrieved and e-mails were sent with a request to distribute the questionnaire among the employed staff. The reminder was sent three times Additionally, individual contacts with university staff were made in order to increase the number of collected responses. Therefore, the collected sample is not a representative sample of the population of the study. 

The survey was anonymous and voluntary. First, each respondent received a written request to participate in the survey together with informed consent The respondents were guaranteed the confidentiality of the data. 

According to the Central Statistical Office [CSO] data, as of 31 December 2020, 93,088 academic professors were employed in Poland. Unfortunately, these statistics lack information about technical and administrative staff. Thus, the size of the population cannot be fully determined. In the study, 732 people participated in the survey, of which 35, or 4.8%, were administrative staff. Thus, the surveyed academic staff constituted 7.5% of the global population. The research results were collected in May and June 2020. 

### 3.2. Data Collection Instrument

The CAWI [Computer Assisted Web Interview] technique was used in this research. The main advantage of the CAWI survey is to provide the respondents with the preferred pace to complete the questionnaire in their natural environment. In the case of the web survey, there is no direct contact with the interviewer so the respondents are more likely to feel more comfortable in providing answers. 

The purpose of the survey was to gather information on perceptions and experiences of remote working in the COVID-19 setting. The questionnaire took approximately 30 min to complete. It consisted of 15 different parts. The present paper reports the results from one part of the study that focused on the aspect of remote working perceptions, competence, training, emotions and perceived control. 

The questionnaire was initially discussed among the researchers and then verified by experts, who suggested some changes in wording. In the next step, a pilot study was conducted on a group of 20 university employees. It allowed identifying possible ambiguities and a further improvement of the questionnaire. After the validation process, the questionnaire was used in the research. In order to ensure reliability, the same question was asked three times, once in identical form, and once reversed. The convergence of responses was 97%. The common method bias was also accounted for to avoid any issues of self-reported data from a single informant. Firstly, by assuring respondents that there were no right or wrong answers and encouraging them to respond as honestly as possible. Secondly, by avoiding ambiguous questions and vague concepts and keeping questions as simple as possible. Thirdly, by grouping the items into sections. The first regarded the characteristics of the respondent. The second regarded experience in remote work, perception of remote work, competencies possessed, participation in training. The third on emotions containing questions, such as I am accompanied by stress resulting from a new situation, I have stress due to unfamiliarity with the tools, I have a constant feeling of uncertainty will I make it on time? I am constantly worried about whether I will be able to organize myself properly, In traditional work settings, I was more satisfied with my work-life balance, Remote work absorbs me more because it takes me much longer to do the same things, My co-workers are more flexible than in traditional settings.

## 4. Results

Of those who participated in the study, 58.2% were women and 41.8% were men; 20% of the respondents were under 40 years of age, respondents aged 41–50 years accounted for 47.5%, respondents aged 51–60 years accounted for 22.1%, and 11.4% of the respondents were over 60 years of age. In terms of employment, 94.8% of the respondents were employed at universities on a full-time basis, and 5.2% represented other forms of employment [self-employment, part-time, contract or commission]; 14.5% of the respondents held managerial positions, that is, they were supervisors for some of the respondents; 21.4% of respondents had work experience of fewer than 10 years, 64.4% of respondents had between 11 and 30 years of work experience and 13.1% of respondents had more than 30 years of work experience. Furthermore, 52.5% of the respondents had to deal with remote work caused by the COVID-19 situation [in the case of teaching and scientific-teaching positions the rate was similar, and lower, about 25%, in the case of administrative staff]. 

**Figure 1:**
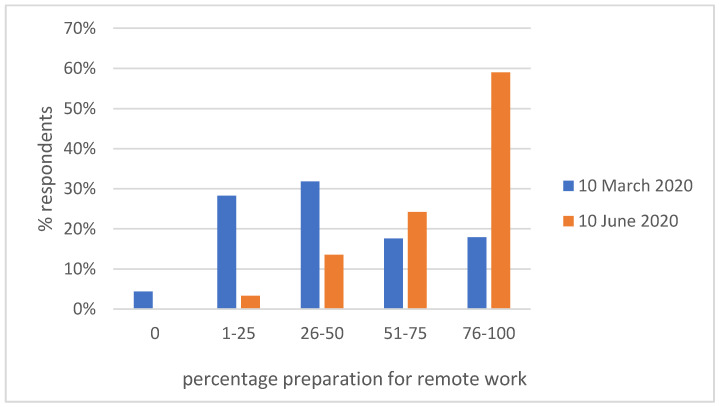
Change in self-assessment of preparation for remote work.

Respondents indicated that their preparation for remote work [Figure 1] changed during the study period. There was a significant increase in the percentage of self-assessment of preparation. 

**Figure 2:**
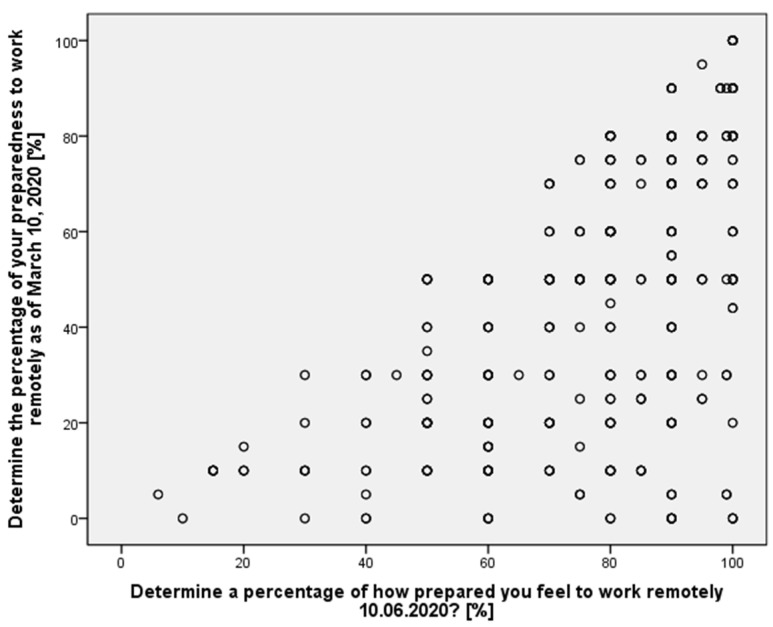
The relationship of perceptions of preparation for remote work before the pandemic and as of the survey date.

The change in perceived competence by position is shown in Figure 2.

The statistics presented in Table 1 indicate that there is a positive significant correlation between the perceived competency to work remotely before and after 3 months of the pandemic period for individual respondents, i.e., all revealed an increase in their competency, and the level of competency in percentage terms was dependent on the starting point. A paired samples test suggests that the perceived level of competence in percentage terms is significantly higher on 10 June 2020 as compared to 10 March 2020. An average of about 30% increase in competence can be found based on the difference in average perceived competence between the periods. The standard deviation across the periods also indicates a significant reduction in the variation of competence perception among the respondents. 

In further analyses [Table 2] we tried to show what the increase in perceived competence depended on. 

What can be seen in Table 2 is that the competency gained over the study period did not depend on either gender, age, seniority, position, or experience in working remotely previous to the COVID-19 pandemic. Competencies possessed at the starting point showed significant differences due to age, position and previous remote work. These results are not surprising since some staff, especially the one involved in research and international cooperation initiatives is accustomed to working remotely, Our research also clearly highlights that crisis conditions offset differences related to remote working competencies in this case. Based on these results, the *Hypothesis 1* was positively verified. There is empirical support to the idea that the incremental competence to work remotely during the initial period of COVID-19 was significant regardless of age, position, and experience.

**Figure 3:**
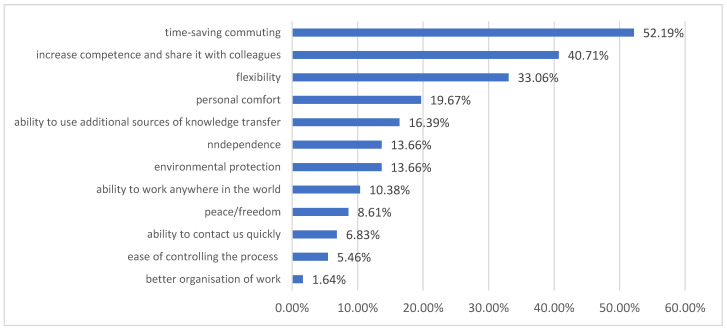
The advantages of remote work indicated by the respondents.

The results of the survey show that the competence gain in question was achieved through participation in external and internal training [35%], self-study [31%] and using both approaches [34%]. Several respondents emphasised the role their employer played in the process of providing means and/or conditions to participate in specialised training. Only 38% of the respondents recalled having participated in external training. The respondents indicated a diversified approach to the training-one of them having participated in as many as 30 trainings in the examined period of 3 months. The majority, however, participated in 2 to 5 trainings. There was a significant change in the approach to remote work. Before the pandemic, Zoom.us, Skype, or Google Drive were used more frequently. In addition, the form of participation in webinars has shifted from viewer to presenter. In the analysed period of time, the most frequently used tools were internal e-learning platforms, MS Teams [the state universities already had received licences for the use of that software], and OneDrive. The advantages of remote work mentioned by the respondents are presented in Figure 3. These were: saving time on commuting [52.19%], raising competence and sharing it with colleagues [40.71%], flexibility [33.06%], personal comfort [19.67%], the ability to use additional sources of transferring knowledge [16.39%], independence [13.66%], environmental protection [13.66%] and the ability to work anywhere in the world [10.38%]. What seems interesting here is the aspect of raising competencies and sharing them with colleagues and the possibility of using additional sources of knowledge transfer. The in-depth research in this area has shown that university employees were able to build teams that shared their experiences and supported others in raising competencies. Employees could draw from such knowledge epicentres. These circles demonstrated that inter-organisational collaboration could produce tangible results for all but did not include the employer boundary, which was an unexpected finding The employees also noticed that diversified materials could be used in remote teaching, which would make the process more appealing and most declared their willingness to use such materials in the future. There were also opinions sustaining that working online allowed getting to know the students better [outside of the classroom context].

**Figure 4:**
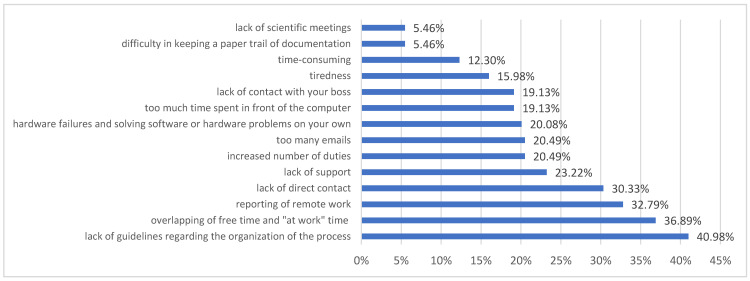
The disadvantages of remote work indicated by the respondents.

The disadvantages of remote work are presented in Figure 4 and comprise the lack of guidelines for the organisation of the process [40.98%], the confusion between free time and at work [36.89%], remote work reporting [32.79%], lack of direct contact [30.33%], lack of support [23.22%], increased number of responsibilities [20.49%], too many emails [20.49%], hardware failures and having to troubleshoot software or hardware problems on one’s own [20.08%], too much time spent in front of the computer [19.13%], lack of contact with the boss [19.13%], and fatigue [15.98%]. Respondents emphasised as well “a lack of opportunity for real discussion, interaction is a serious limitation”. From an organisational point of view, the universities were deficient in managerial competencies related to change management as processes had been implemented. Employees felt a lack of guidance and a lack of support from the superiors and the institution. Emotional conditions were also related to these aspects and analysed here in terms of fear, uncertainty, and a sense of dissonance. 

To assess negative emotions linked to remote work, respondents were asked the questions presented in Table 3. The descriptive statistics point to high levels of stress and uncertainty among respondents and suggest a wide variation in the experiences.

The results show that there was no variation in perceived emotions based on gender, age, position, work experience and previous experience of remote work. The calculated Cronbach’s alpha coefficient of 0.884 for these proposed four questions indicates the reliability of the scale and the exploratory factor analysis indicates its homogeneity, provided that this factor explains 74.13% of the variability.

Based on these results the *Hypothesis 2* was validated. More specifically, employees did experience negative emotions during the period of study in which they were confronted with a new situation, had to deal with several unexpected situations among which unfamiliarity with the tools, and experienced high levels of uncertainty.

To show the emotional dissonance of remote work versus regular work, three questions were asked as shown in Table 4.

The results indicate that work-life balance is the least satisfying among respondents. However, it is important to remember that it takes time to learn or stabilize new situations and the respondents of this study did not have that time. Only subsequent surveys may show if and how has such dissonance been normalised. Surprisingly, the respondents felt that their colleagues were more flexible than in traditional conditions, which may be due to the fact that people tend to unite in crisis situations. *Hypothesis 3* stated that employees experienced emotional dissonance during the study period which was verified based on the results.

## 5. Discussion

The pandemic has shown that remote work is possible even in the areas previously associated with traditional work [4]. A large number of workers had to incorporate technology into their daily routine [1]. In higher education institutions, video conferencing solutions, such as MS Teams, Skype or Zoom, started to be widely and successfully used by millions of users-both university staff and students [3]. The significant increase in digital competencies among the teaching staff and students and the range to which these competencies were found applicable have allowed remote work to become a “new normal” [18]. The most important determinants affecting the assessment of remote work include the perceived level of self-efficacy, the assessment of the level of social support perceived by the employee that was received from the supervisor and colleagues, and the degree of interdependence of the performed tasks [67,68]. 

Both the advantages and disadvantages of remote work considered three levels of analysis: personal [experienced by employees], organisational and social [30]. The results of the positive and negative effects of remote work clearly suggest its duality. Several aspects discussed previously in the literature [13,69] and included in the present study may constitute an advantage or a limitation. For example, the lack of the need to work within fixed hours may, on the one hand, contribute to reducing the stress of an employee and increasing their productivity, and on the other hand, cause extended working time and a loss of work-life balance [1,70,71,72]. Similarly, the possibility of working from home may foster the integration of professional and caring responsibilities and, as a result, reduce travel costs to work [9,23]. On the other hand, however, the constant availability of an employee may weaken this balance and cause tension in family relationships [59,73,74]. Technology related to remote work can also increase employees’ skills, but, at the same time, cause uncertainty and work-related stress [10,59]. Perhaps the most important personal aspect of this research is savings on commuting. That is confirmed by the reduced time [10] and costs of commuting to work by car or public transportation [3,9,23,30,59,75]. Technology creates ways to overcome distance [30]. Our research lacked the organisational aspect indicated in other studies, related to the ability to better manage the time [70,76].On the other hand, the respondents paid attention to the social aspect characterised by the reduction of traffic jams, energy consumption and pollution of the environment as a result of fewer employees traveling to work [30].

It is important to show the negative emotional feelings [fears] of employees related to the transition to an online mode [9,76]. This mainly includes fear and stress caused by a new situation, unfamiliarity with the tools, a sense of wasting time and a constant worry about self-organisation. In this situation, university employees are accompanied by emotional dissonance that affects the emotional well-being of employees, especially in a pandemic [30].

The disadvantages of remote work on the personal level regard specifically the increase of the time spent working [30] with a negative impact on the perception of work-life balance. Our research adds to that some other factors that include social isolation with limited contact with colleagues [3,13], no possibility to drink coffee or talk to colleagues, the loss of informal communication and the feeling of loneliness [30]. Mental isolation causes the perception of being separated from others [10], failure to meet the need for support and understanding, and other emotional aspects of interpersonal interaction [26,77]. Sharing knowledge and skills and searching for solutions to emerging problems together are considered positive aspects by university employees. Conversely, another finding has suggested that isolation can promote a feeling of integration when the ever-present technology is in place [78]. It can be seen in the form of self-monitoring and being interested in the problems of a co-worker. Our research also emphasises the aspect of the organisational disadvantage of remote work in the form of a loss of management control [30] highlighted by difficulties in supervising and communicating with employees [13]. This is confirmed by poor change management and the lack of diagnosis required in incidental situations. The respondents emphasised that their organisation seemed only concerned with support in the form of training in digital competencies forgetting other areas of possible intervention. 

The analysis of the remote work experiences would be incomplete if it comprised only the positive and negative aspects without reflecting on its impact on an individual level. The urgent transition to a new way of working could not have no impact on the emotional aspects of human existence and the perception of subjective wellbeing. Not surprisingly, surveyed individuals did feel the difference in that regard when comparing the traditional and remote work settings. The new way of doing things resulted also in more time needed to do the same things. The results show that high levels of stress were experienced particularly by those who were attached to electronic devices and constantly online [78], a behaviour that the pandemic has promoted. Emotional dissonance in the remote working environment is related to how remote and traditional work is perceived. Understanding and responding to the specificity of work context is of utmost importance as research has linked it to the effects on performance and wellbeing [67,77].

Our research advances the understanding of digitalised workplaces, and the ways in which they affect emotions and fills a gap in the area of research on the role of emotions in the digital workplace [79]. We believe that with increased digitalisation, tested against the recent pandemic situation, we have shown the importance of emotions and the ways they emerge, are expressed, shared, or suppressed within the digitalised work-related context. 

The results suggest that in the introduction of new technology at work the psychological dimension is very fundamental and should not be dismissed [80]. Therefore, humanistic objectives should not be lost in any of the information systems used [81]. 

## 6. Implications and Limitations

This study examined how independent external changes determine the growth of competence in employees, in this case, to working remotely. Working in conditions that are difficult for everyone forces employees to cooperate, even across company boundaries, and increase each other’s competencies. Situations of this type also increase the reciprocity of flexibility of colleagues. Undertaking changes and their implementation, as research has shown, requires a significant and different commitment from the side of the management team. In these types of situations, employees require more frequent contact and interest from the management. New situations and especially crisis situations intensify negative emotions and emotional dissonance. 

The study was limited by a lack of representativeness of the sample, despite its considerable size, it is not possible to generalise the results. It is recommended that further research be conducted with a more representative sample because a full survey as shown was not possible. Thus, it is worth paying attention to this in the next research in order to make the research replicable. It is also worth repeating the research after the COVID-19 period and doing a comparative analysis of the changes that occurred. We are aware of the disadvantages of the proposed approach, but we wanted to emphasise the impact of the current one on the implementation and management of change. Despite the above limitations, the results of this study show the importance of emotions in remote work. Authors should discuss the results and how they can be interpreted from the perspective of previous studies and of the working hypotheses. The findings and their implications should be discussed in the broadest context possible. Future research directions may also be highlighted.

## 7. Conclusions

The article is a combination of the current approach and knowledge to issues related to remote work. gives them a new meaning and entails a number of new challenges. Although the issue of remote work has been discussed in previous research, the analysis of the literature shows a negligible number of studies that would discuss it in the context of a situation of coercion for the employee and the associated emotional conditions. The pressure resulting from a sudden and unpredictable change of the current conditions and the need to acquire a number of new skills in a very short time seem to significantly change the attitude of employees towards remote work.

The considerations presented in this study suggest that independent external determinants significantly affected the remote work competencies acquired by employees and organisations in crisis. An important finding is that these determinants are independent of age, position, work experience, or prior remote work experience. It is evident that higher education institutions need now to recognise and work on the change management competencies of the management staff in order to be better prepared for facing the externally-induced challenges. It is interesting to note that the emotions associated with uncertainty or a new situation have also been found independent of age, position, work experience, or prior remote work experience.

Emotions that accompanied employees during the crisis may determine the level of acceptance and the dynamics of further development of remote work in the post-pandemic period. The conducted research was the initial stage of research on remote work issues as a consequence of the COVID-19 pandemic, linking them with the employee’s emotional sphere.

## Figures and Tables

**Table 1:** Statistics for determining the percentage of respondents’ preparedness to work remotely.

Determine the Percentage of Your Preparedness to Work Remotely [%]		
Day	10 March 2020	10 June 2020
Mean	45.05	75.77
Std. Deviation	27.944	20.793
Paired Samples Correlations	0.585 [*p* < 0.0001]	
Paired Samples Test	T = −35.969179 [*p* < 0.0001]	

**Table 2:** Test for Equality.

Categories	N	Mean	Std. Deviation	Test for Equality of Variances		Test for Equality of Means	
				*F*	*p*	*t*	*p*
Gender ^1^							
female	424	30.1108	23.13021	0.003	0.957	−0.835	0.404
male	308	31.5552	23.08419				
Age ^2^							
Under 30 years	24	27.7083	23.12439	0.961	0.428	0.276	0.894
30–40 years	122	31.6311	23.71846				
40–50 years	345	30.9652	22.88975				
50–60 years	165	29.6061	21.82991				
Over 60 years	76	31.5000	26.05763				
Length of service ^2^							
Under 10 years	157	30.9427	23.81398	0.263	0.769	1.022	0.360
10–30 years	479	31.2714	23.00167				
Over 30 years	96	27.5938	22.43474				
Position ^2^							
administrative employee	26	35.1538	24.21189	0.666	0.514	0.782	0.458
teaching	107	32.0748	23.90378				
research and teaching associate	416	30.1851	22.62107				
previous remote working experience ^1^							
yes	384	30.4531	23.08838	0.008	0.928	−0.326	0.744
no	348	31.0115	23.15528				

^1^ Levene’s and *t*-test. ^2^ Test of Homogeneity of Variances and ANOVA.

**Table 3:** Emotions associated with working conditions.

Question		I Am Accompanied by Stress Resulting from a New Situation	I Have Stress Due to Unfamiliarity with the Tools	I Have a Constant Feeling of Uncertainty-Will I Make It on Time?	I Am Constantly Worried about whether I Will be able to Organize Myself Properly
Mean		6.86	6.33	6.15	6.01
Std. Deviation		2.029	1.888	1.963	1.992
Gender ^1^	*t*	0.477	1.137	0.430	1.757
	*p*	0.634	0.256	0.667	0.079
Age ^2^	*t*	0.395	1.620	0.256	0.880
	*p*	0.812	0.167	0.906	0.476
Length of service ^2^	*t*	0.843	0.387	1.497	2.877
	*p*	0.431	0.679	0.224	0.057
Position ^2^	*t*	1.743	0.369	1.172	1.759
	*p*	0.176	0.691	0.310	0.173
Previous remote working experience ^1^	*t*	−0.202	1.215	−1.120	−0.338
	*p*	0.840	0.225	0.263	0.736

^1^ Levene’s and *t*-test. ^2^ Test of Homogeneity of Variances and ANOVA.

**Table 4:** Descriptive Statistics.

Question	Mean	Std. Deviation
In traditional work settings, I was more satisfied with my work-life balance	5.76	1.790
Remote work absorbs me more because it takes me much longer to do the same things.	4.75	2.042
My co-workers are more flexible than in traditional settings	5.57	1.640

## Data Availability

The dataset supporting the conclusions of this article is included within the article. A copy of the questionnaire and data can be obtained from the first author.
